# Comprehensive analysis of PDE2A: a novel biomarker for prognostic value and immunotherapeutic potential in human cancers

**DOI:** 10.1590/1414-431X2024e14220

**Published:** 2024-12-13

**Authors:** Zhen Yu, Yawen Song, Jin Wang, Yujing Wu, Hefang Wang, Shuye Liu, Yu Zhu

**Affiliations:** 1 Nankai University Affinity the Third Central Hospital, Tianjin Third Central Hospital, Tianjin, China; 2 Qingpu Branch of Zhongshan Hospital Affiliated to Fudan University, Shanghai, China; 3 College of Chemistry, Nankai University, Tianjin, China

**Keywords:** Phosphodiesterase 2A (PDE2A), Comprehensive analysis, Gene expression, Prognostic marker, Immunotherapy target

## Abstract

Phosphodiesterase 2A (PDE2A) plays a pivotal role in modulating cyclic nucleotide metabolism. Recent studies have shown that PDE2A is associated with some tumors, but its expression profiles, prognostic significance, and immunological roles in diverse cancer types remain unclear. Utilizing advanced bioinformatics tools, we performed a comprehensive analysis of PDE2A gene expression in multiple human cancers. Our study revealed that PDE2A expression was significantly reduced in the majority of cancer types and clinicopathological stages (I to IV) compared to normal tissues. Additionally, PDE2A expression was closely related to the prognosis of cancers such as stomach adenocarcinoma (STAD), ovarian serous cystadenocarcinoma (OV), and liver hepatocellular carcinoma (LIHC). Cox regression analyses indicated that PDE2A can act as an independent prognostic factor for these cancers. The level of PDE2A DNA methylation was significantly decreased in most cancers. Genetic alterations in PDE2A predominantly manifest in the form of amplifications. Moreover, infiltrating cells and immune checkpoint genes, including PDCD1, exhibited notable correlations with PDE2A expression. Significant associations were observed between PDE2A expression and tumor mutation burden as well as microsatellite instability. Single cell sequencing revealed PDE2A's crucial role in regulating differentiation and angiogenesis of cancer cells. Functional enrichment analysis emphasized the important role of PDE2A in synaptic transmission and tumor development. Aberrant expression of PDE2A influenced the sensitivity of various antitumor and chemotherapy drugs. This research provided a comprehensive analysis of PDE2A in human cancers, highlighting its potential as both a prognostic marker and an immunotherapy target for future research.

## Introduction

Cancer, a multifaceted and heterogeneous disease, profoundly impacts human health and quality of life (1). In recent years, pan-cancer analysis has emerged as a valuable approach for exploring innovative tumor therapies (2). It involves comprehensive studies encompassing multiple cancer types to identify the commonalities and differences in their genetic, epigenetic, and molecular characteristic (3). This analysis has the potential to reveal new biomarkers and therapeutic targets, paving the way for more personalized and effective cancer treatment.

Phosphodiesterase 2A (PDE2A) is a member of the phosphodiesterases (PDEs) superfamily (4). PDEs are a diverse group of enzymes that regulate cellular signaling by modulating cyclic nucleotide levels (5). PDE2A specifically hydrolyzes cyclic adenosine monophosphate (cAMP) and cyclic guanosine monophosphate (cGMP), thereby influencing important intracellular signaling pathways (6). Aberrant expression or activity of PDE2A has been associated with the dysregulation of cellular processes such as proliferation, migration, invasion, and angiogenesis, which are pivotal hallmarks of cancer progression (7-10). Chen et al. (7) found that overexpression of PDE2A could inhibit the proliferation, colony formation, migration, and invasion of hepatocellular carcinoma cells by regulating ATP content and mitochondrial morphology. In osteosarcoma, Murata et al. (8) revealed that cell proliferation and cell migration were regulated by PDE2A-cAMP signaling and PDE2A-cGMP signaling, respectively. The study conducted by Li et al. (9) demonstrated that PDE2A overexpression decelerated glioma progression through the suppression of cAMP accumulation and GSK-3β phosphorylation. However, the exact role of PDE2A in cancer has not yet been fully elucidated. Therefore, it is necessary to explore the expression patterns and functional roles of PDE2A in different cancer types to gain a comprehensive understanding of its involvement in tumorigenesis and progression.

In this study, we undertook a comprehensive pan-cancer analysis of PDE2A expression, aiming to decipher its differential expression patterns across diverse human cancers and correlate these patterns with clinicopathological features, prognostic outcomes, genetic alterations, immunological functions, and drug sensitivity. Our findings may provide novel insights into the complex roles of PDE2A in cancer biology and highlight its potential as a prognostic and immunotherapeutic target in multiple cancer types.

## Material and Methods

### Gene expression analysis

The expression profiles of PDE2A across various cancer types and adjacent normal tissues were assessed using TIMER2.0 (http://timer.cistrome.org/, accessed on 10 April 2024). Gene expression levels were represented on a log2 (TPM + 1) scale, with TPM denoting transcripts per million. In cases where TIMER2.0 lacked adjacent normal samples for certain tumor tissue, we integrated data from The Cancer Genome Atlas (TCGA) database with Genotype-Tissue Expression (GTEx) information via GEPIA2.0 (http://gepia2.cancer-pku.cn/, accessed on 10 April 2024). This approach allowed for a more comprehensive and robust comparative analysis of gene expression between normal and tumor tissue. Furthermore, protein expression patterns of PDE2A across diverse cancer types were investigated utilizing the Clinical Proteomic Tumor Analysis Consortium (CPTAC) from the University of Alabama Birmingham Cancer (UALCAN) data analysis portal database (http://ualcan.path.uab.edu/analysis-prot.html, accessed on 12 April 2024). In addition, we explored the relationship between PDE2A expression and different tumor stages (stages I to IV) using UALCAN.

### Survival prognosis analysis

Prognostic analysis of overall survival (OS) and disease-free survival (DFS) was performed using “Survival Analysis” module via GEPIA2.0 tool (accessed on 13 April 2024). We set the parameter Group Cutoff to “Median” and categorized patients into high-expression group and low-expression group based on the cut-off values with parameters Cutoff-High=50% and Cutoff-Low=50%. Moreover, Kaplan-Meier plotter and survival maps were generated, with calculation of log-rank P-value and hazard ratio (HR). To investigate the correlation between PDE2A expression and clinical characteristics of tumor patients, we conducted univariate and multivariate Cox regression analyses using R packages (version 4.2.1, Austria). A characteristic was considered an independent prognostic factor if its P-value was less than 0.05 in both analyses.

### Analysis of DNA methylation

Using the UALCAN tool (accessed on 15 April 2024), we compared the DNA methylation patterns of PDE2A in various cancer tissues with those in normal tissues.

### Genetic alteration analysis

The genomic location of the PDE2A gene was retrieved from the GeneCards website (https://www.genecards.org/, accessed on 20 April 2024). Comprehensive analysis of PDE2A mutations in 10967 samples from TCGA database was conducted using the cBioPortal (http://www.cbioportal.org, accessed on 22 April 2024), with exploration of genetic alteration characteristics. Modules such as “Cancer Type Summary” and “Mutations” were utilized.

### Immune cell infiltration and tumor microenvironment (TME) analysis

Immune cell infiltration analysis was conducted via the Sangerbox tool (http://sangerbox.com/, accessed on 18 July 2024). The heatmap of the association between PDE2A expression and immune cell infiltration in different TCGA cancers was obtained by using quanTIseq algorithm (11). Spearman's correlation coefficient was calculated. The stromal and immune cells in malignant tumor microenvironment was evaluated by the ESTIMATE (12). It employs single-sample Gene Set Enrichment Analysis to generate three scores: stromal score, which quantifies the presence of stroma in the tumor tissue; immune score, reflecting the infiltration of immune cells in the tumor; and estimate score, which infers the purity of the tumor. We calculated the correlation between PDE2A expression levels and these three scores using the Sangerbox platform (accessed on 23 April 2024).

### Correlation of PDE2A with immune checkpoint (ICP) genes, tumor mutation burden (TMB), and microsatellite instability (MSI)

Correlations between PDE2A and ICP genes, TMB and MSI were analyzed separately based on the SangerBox portal (accessed on 26 April 2024) using TCGA data (13). Sixty ICP genes included 24 inhibitory genes and 36 stimulatory genes were selected (14). Spearman's correlation analysis was applied to get the correlation coefficient.

### Single cell sequencing analysis

CancerSEA database (http://biocc.hrbmu.edu.cn/CancerSEA/; accessed on 28 April 2024) was used to investigate the functional states of cancer cells at the single cell level (15). Correlation data between PDE2A expression and different tumor functional specificity based on single-cell RNA sequencing were explored. We gained t-distributed stochastic neighbor embedding (T-SNE) diagrams of individual cells using CancerSEA tool.

### Gene enrichment analysis

The BioGRID website (https://thebiogrid.org/; accessed on 8 May 2024) was used to obtain the protein-protein interaction network (16). GEPIA2.0 (accessed on 8 May 2024) was used to download the top 100 PDE2A-correlated genes from TCGA data. A pairwise gene-gene Pearson's correlation analysis between PDE2A and the top four correlated genes was conducted using GEPIA2.0. Moreover, we generated a heatmap to show the correlations between PDE2A and the top four genes from TIMER2.0 database (accessed on 8 May 2024). A Gene Ontology (GO) enrichment analysis and a Kyoto encyclopedia of genes and genome (KEGG) pathway analysis were performed by DAVID (17). The results were visualized through the Sangerbox tool.

### Drug sensitivity of PDE2A in pan-cancer

RNAactDrug website (http://bio-bigdata.hrbmu.edu.cn/RNAactDrug/index.jsp; accessed on 10 May 2024) offered us a valuable condition to explore the correlation between drug sensitivity and RNA molecules (18^18^). We entered “PDE2A” in the “RNA molecule” and chose “Expression” in “Omics” to perform the drug sensitivity of PDE2A in pan-cancer. Spearman's analysis was used to identify the top five drugs with significant positive correlation and the top five drugs with significant negative correlation (19).

### Statistical analysis

The significance of PDE2A expression levels between tumor and normal tissues was assessed using the Wilcoxon rank-sum test for comparison. Prognostic analysis was conducted utilizing the Kaplan-Meier method (log-rank test) and univariate/multivariate Cox regression analyses. Additionally, a Spearman correlation analysis was performed to evaluate the association between PDE2A and other factors, including immune cell infiltration, TME, TMB, and MSI. The statistical significance of the aforementioned analyses was determined when the P value <0.05.

## Results

### Differential expression patterns of PDE2A in human cancers

**Figure 1:**
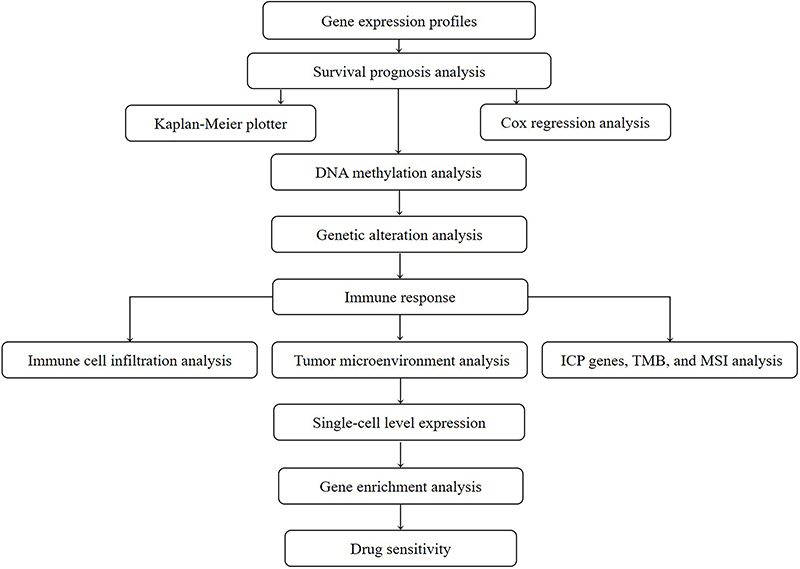
Study workflow. ICP: immune checkpoint; TMB: tumor mutation burden; MSI: microsatellite instability.

**Figure 2:**
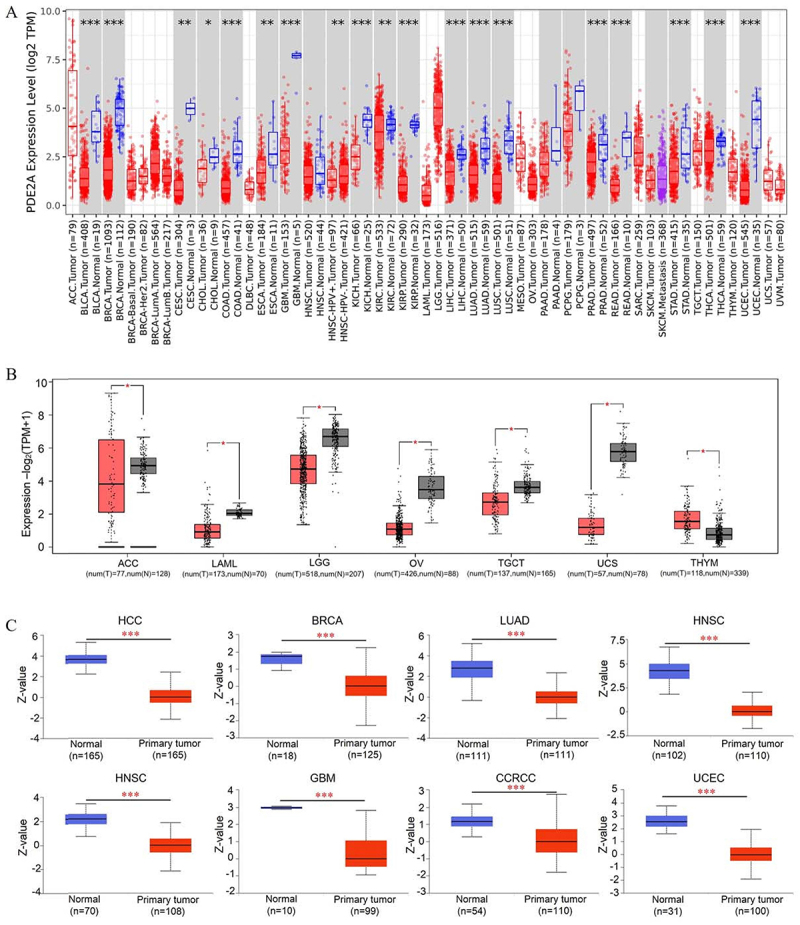
The expression status of PDE2A in pan-cancer. **A**, Differential expression levels of PDE2A between tumor samples and paired normal tissues across various cancers in the TCGA database (*P<0.05, **P<0.01, ***P<0.001). **B**, Comparative analysis of PDE2A expression differences among ACC, LAML, LGG, OV, TGCT, UCS, and THYM using data from Genotype-Tissue Expression and The Cancer Genome Atlas (*P<0.05). **C**, Assessment of PDE2A protein expression levels in primary tissues *vs* normal tissues for HCC, BRCA, LUAD, LUSC, HNSC, GBM, CCRCC, and UCEC based on the CPTAC dataset (***P<0.001). Lines in boxes represent median and interquartile range (Wilcoxon rank-sum test).

The workflow of our study is illustrated in Figure 1. Utilizing data from the TCGA project, we constructed a comprehensive overview of PDE2A expression across various human cancers. Figure 2A illustrates notable variations in the transcriptional levels of PDE2A between tumor and adjacent normal tissues across 18 distinct cancer types. Specifically, decreased expression of PDE2A was observed in bladder urothelial carcinoma (BLCA), breast carcinoma (BRCA), cervical squamous cell carcinoma and endocervical adenocarcinoma (CESC), cholangiocarcinoma (CHOL), colon adenocarcinoma (COAD), esophageal carcinoma (ESCA), glioblastoma multiforme (GBM), kidney chromophobe (KICH), kidney renal clear cell carcinoma (KIRC), kidney renal papillary cell carcinoma (KIRP), liver hepatocellular carcinoma (LIHC), lung adenocarcinoma (LUAD), lung squamous cell carcinoma (LUSC), prostate adenocarcinoma (PRAD), rectum adenocarcinoma (READ), stomach adenocarcinoma (STAD), thyroid carcinoma (THCA), and uterine corpus endometrial carcinoma (UCEC) compared to their respective normal counterparts (P<0.05).

To enhance the breadth of our analysis, we integrated data from the GTEx datasets, bolstering the number of normal tissue samples serving as controls in the TCGA project. As depicted in Figure 2B, PDE2A levels were notably lower in adrenocortical carcinoma (ACC), acute myeloid leukemia (LAML), lower-grade glioma (LGG), ovarian serous cystadenocarcinoma (OV), testicular germ cell tumors (TGCT), and uterine carcinosarcoma (UCS) tissues relative to their corresponding normal tissues. Conversely, in thymoma (THYM) tissues, PDE2A levels were significantly elevated compared to normal tissues (P<0.05).

Furthermore, leveraging the CPTAC datasets, we investigated the differences in PDE2A protein expression between tumor and corresponding normal tissues. Our findings revealed significant downregulation of PDE2A protein levels in hepatocellular carcinoma (HCC), BRCA, LUAD, LUSC, head and neck squamous cell carcinoma (HNSC), GBM, clear cell renal cell carcinoma (CCRCC) and UCEC, as shown in Figure 2C.

### Pancancer analysis of the connection between PDE2A expression and clinicopathology

**Figure 3:**
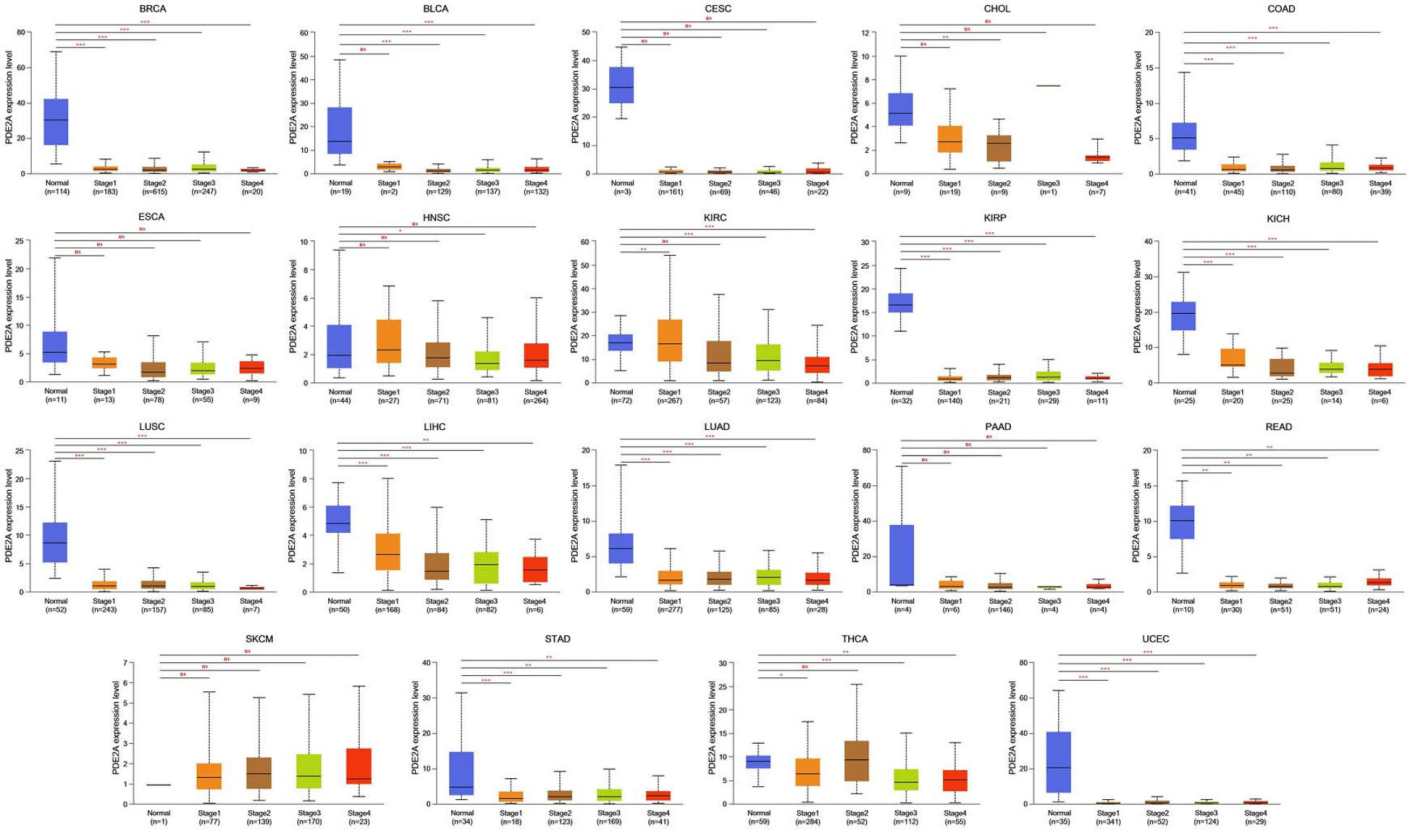
Correlation between PDE2A gene expression and different pathological stages of BRCA, BLCA, CESC, CHOL, COAD, ESCA, HNSC, KIRC, KIRP, KICH, LUSC, LIHC, LUAD, PAAD, READ, SKCM, STAD, THCA, and UCEC based on The Cancer Genome Atlas dataset (*P<0.05, **P<0.01, ***P<0.001, ns: no significance). Lines in boxes represent median and interquartile range (Student's*t*-test).

In order to investigate the correlation between PDE2A expression and clinical pathological characteristics, we employed the UALCAN platform to analyze the variations in PDE2A expression across normal tissues and tumor tissues at different stages of cancer development. As illustrated in Figure 3, a significant downregulation of PDE2A expression was confirmed in a spectrum of cancers, including BRCA, BLCA, COAD, KIRP, KICH, LUSC, LIHC, LUAD, READ, STAD, and UCEC, from normal tissues to early malignant tumors and further into advanced stages. However, in several cancers such as CESC, ESCA, CHOL, HNSC, pancreatic adenocarcinoma (PAAD), and skin cutaneous melanoma (SKCM), there was no statistically difference in PDE2A expression throughout the progression of tumors.

### The prognostic value of PDE2A

**Figure 4:**
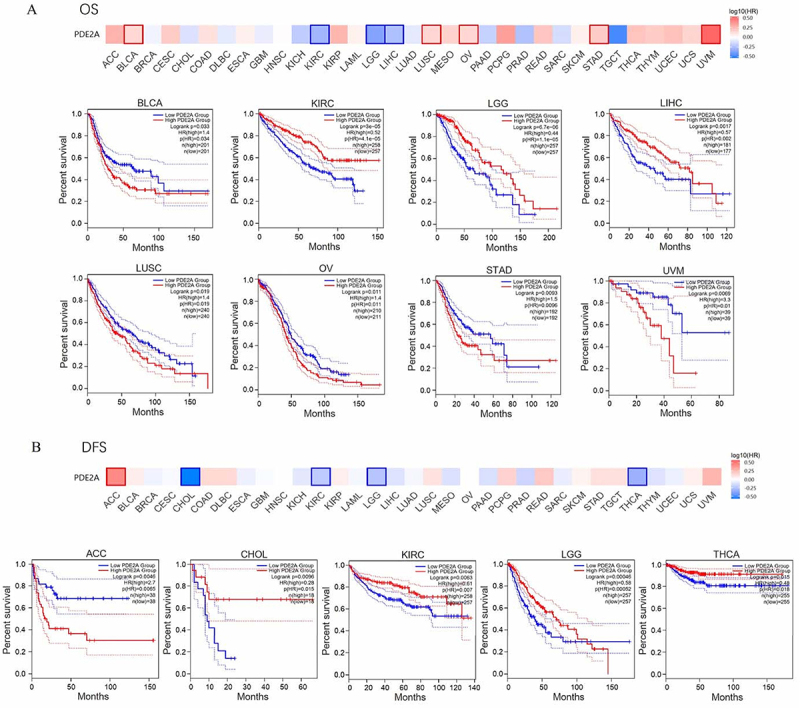
Prognostic values of PDE2A gene expression in pan-cancerous tumors. **A**, Correlation of overall survival (OS) with PDE2A gene expression in BLCA, KIRC, LGG, LIHC, LUSC, OV, STAD, and UVM. **B**, Correlation of disease-free survival (DFS) with PDE2A gene expression in ACC, CHOL, KIRC, LGG, and THCA.

We employed the GEPIA2.0 tool to assess the prognostic significance of PDE2A expression in cancer patients. OS analysis showed that high PDE2A expression group was significantly associated with good prognosis in KIRC (P=4.1e-05), LGG (P=1.1e-05), and LIHC (P=0.002). However, highly expressed PDE2A group was related to poor prognosis in BLCA (P=0.034), LUSC (P=0.019), OV (P=0.011), STAD (P=0.0096), and uveal melanoma (UVM) (P=0.01) (Figure 4A). Figure 4B demonstrates that the reduced PDE2A expression group was associated with favorable DFS in ACC (P=0.0065). In contrast, enhanced expression of PDE2A was related to good DFS in CHOL (P=0.015), KIRC (P=0.007), LGG (P= 0.00052), and THCA (P=0.018). These results suggested that PDE2A may be regarded as a potential prognostic biomarker for many tumors.

To further evaluate whether the expression of PDE2A serves as a prognostic factor for cancer patients independently of other clinical characteristics (age, gender, pathological stage, histologic grade, and race), we conducted univariate and multivariate Cox regression analyses in patients with STAD, OV, and LIHC. The results suggested that age >65 (HR=2.019, 95%CI: 1.403-2.906, P<0.001), Stage III (HR=2.280, 95%CI: 1.201-4.329, P=0.012), Stage IV (HR=5.475, 95%CI: 2.641-11.349, P<0.001), and PDE2A expression (HR=1.788, 95%CI: 1.258-2.540, P=0.001) were independent prognostic factors in STAD (Table 1). In OV patients, age >60 (HR=1.456, 95%CI: 1.118-1.896, P=0.005), Stage II (HR =0.081, 95%CI: 0.009-0.722, P=0.024), and PDE2A expression (HR =1.358, 95%CI: 1.043-1.767, P=0.023) were independent factors for prognosis (Table 2). Further, Stage III (HR=2.467, 95%CI: 1.611-3.777, P<0.001), Stage IV (HR=5.173, 95%CI: 1.593-16.805, P=0.006), and PDE2A expression (HR=0.449, 95%CI: 0.305-0.660, P<0.001) were independent prognostic variables in LIHC (Table 3). Overall, these results showed that PDE2A could be an independent predictor of poor outcome in STAD and OV patients, but of good prognosis in LIHC patients.

**Table 1:** Univariate/multivariate Cox regression analysis of clinicopathological characteristics of stomach adenocarcinoma associated with overall survival.

	Total (n)	Univariate analysis		Multivariate analysis	
		Hazard ratio (95%CI)	P value	Hazard ratio (95%CI)	P value
Age					
≤65	163	Reference		Reference	
>65	204	1.620 (1.154-2.276)	**0.005**	2.019 (1.403-2.906)	**<0.001**
Gender					
Female	133	Reference			
Male	237	1.267 (0.891-1.804)	0.188		
Pathologic stage					
Stage I	50	Reference		Reference	
Stage II	110	1.551 (0.782-3.078)	0.209	1.647 (0.828-3.274)	0.155
Stage III	149	2.381 (1.256-4.515)	**0.008**	2.280 (1.201-4.329)	**0.012**
Stage IV	38	3.991 (1.944-8.192)	**<0.001**	5.475 (2.641-11.349)	**<0.001**
Histologic grade					
G1	10	Reference			
G2	134	1.648 (0.400-6.787)	0.489		
G3	217	2.174 (0.535-8.832)	0.278		
Race					
Asian/Black or African American	84	Reference			
White	236	1.248 (0.802-1.943)	0.326		
PDE2A					
Low	185	Reference		Reference	
High	185	1.604 (1.151-2.235)	**0.005**	1.788 (1.258-2.540)	**0.001**

Bold type indicates statistically significant.

**Table 2 t02:** Univariate/multivariate Cox regression analysis of clinicopathological characteristics of ovarian serous cystadenocarcinoma associated with overall survival.

	Total (n)	Univariate analysis		Multivariate analysis	
		Hazard ratio (95%CI)	P value	Hazard ratio (95%CI)	P value
Age					
≤60	207	Reference		Reference	
>60	172	1.352 (1.045-1.749)	**0.022**	1.456 (1.118-1.896)	**0.005**
Clinical stage					
Stage I	1	Reference		Reference	
Stage II	23	0.080 (0.009-0.688)	**0.021**	0.081 (0.009-0.722)	**0.024**
Stage III	294	0.193 (0.027-1.397)	0.103	0.216 (0.028-1.648)	0.140
Stage IV	58	0.240 (0.033-1.766)	0.161	0.281 (0.036-2.179)	0.225
Histologic grade					
G1&G2	46	Reference			
G3&G4	323	1.239 (0.838-1.833)	0.283		
Race					
Asian/Black or African American	37	Reference		Reference	
White	329	0.644 (0.409-1.014)	0.058	0.601 (0.377-0.957)	**0.032**
PDE2A					
Low	188	Reference		Reference	
High	191	1.340 (1.035-1.735)	**0.026**	1.358 (1.043-1.767)	**0.023**

Bold type indicates statistically significant.

**Table 3:** Univariate/multivariate Cox regression analysis of clinicopathological characteristics of liver hepatocellular carcinoma associated with overall survival.

	Total (n)	Univariate analysis		Multivariate analysis	
		Hazard ratio (95%CI)	P value	Hazard ratio (95%CI)	P value
Age					
≤60	177	Reference			
>60	196	1.205 (0.850-1.708)	0.295		
Gender					
Female	121	Reference			
Male	252	0.793 (0.557-1.130)	0.200		
Pathologic stage					
Stage I	173	Reference		Reference	
Stage II	86	1.417 (0.868-2.312)	0.164	1.209 (0.737-1.984)	0.452
Stage III	85	2.734 (1.792-4.172)	**<0.001**	2.467 (1.611-3.777)	**<0.001**
Stage IV	5	5.597 (1.726-18.148)	**0.004**	5.173 (1.593-16.805)	**0.006**
Histologic grade					
G1	55	Reference			
G2	178	1.162 (0.686-1.969)	0.576		
G3	123	1.185 (0.683-2.057)	0.545		
G4	12	1.681 (0.621-4.549)	0.307		
Race					
Asian/Black or African American	176	Reference			
White	185	1.265 (0.881-1.816)	0.203		
PDE2A					
Low	186	Reference		Reference	
High	187	0.440 (0.308-0.628)	**<0.001**	0.449 (0.305-0.660)	**<0.001**

Bold type indicates statistically significant.

### Pan-cancer analysis of DNA methylation patterns of PDE2A

**Figure 5:**
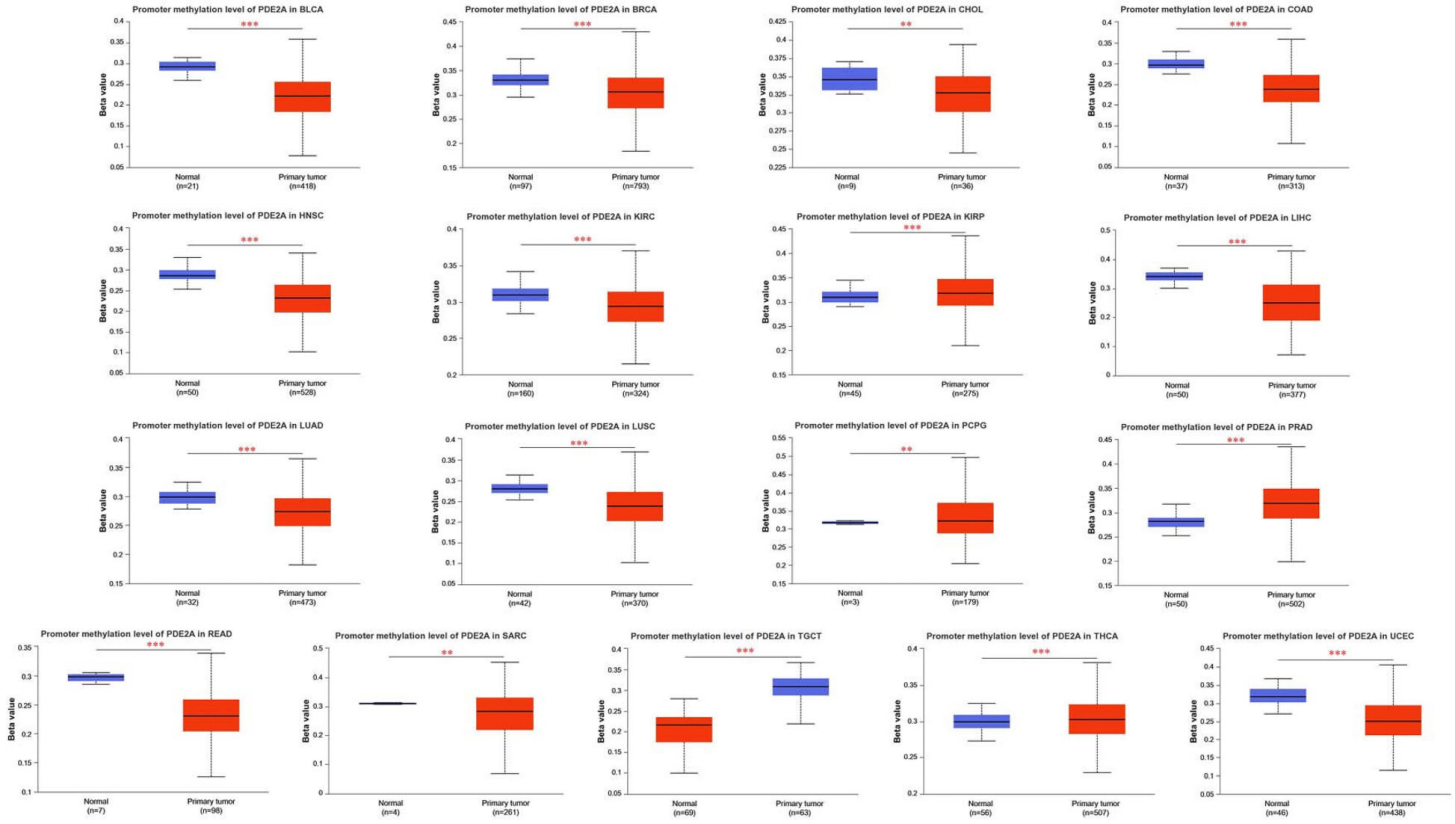
Promoter methylation levels of PDE2A in various cancer types. The UALCAN tool was employed to analyze and compare the methylation values of PDE2A between normal and primary tumor tissues. *P<0.05, **P<0.01, ***P<0.001. Lines in boxes represent median and interquartile range (Student's*t*-test).

Aberrant DNA methylation has been implicated in increasing the susceptibility to cancer development (20). Leveraging the TCGA project, we employed UALCAN to investigate the potential association between the methylation level of PDE2A and tumorigenesis. Our findings revealed a significant increase in the methylation of PDE2A in specific tumor tissues, such as KIRP, pheochromocytoma and paraganglioma (PCPG), PRAD, TGCT, and THCA. Conversely, we observed a notable decline in the methylation level of PDE2A in a range of cancer types, including BLCA, BRCA, CHOL, COAD, HNSC, KIRC, LIHC, LUAD, LUSC, READ, SARC, TGCT, and UCEC compared to normal tissues (Figure 5).

### Genetic alteration of PDE2A

**Figure 6:**
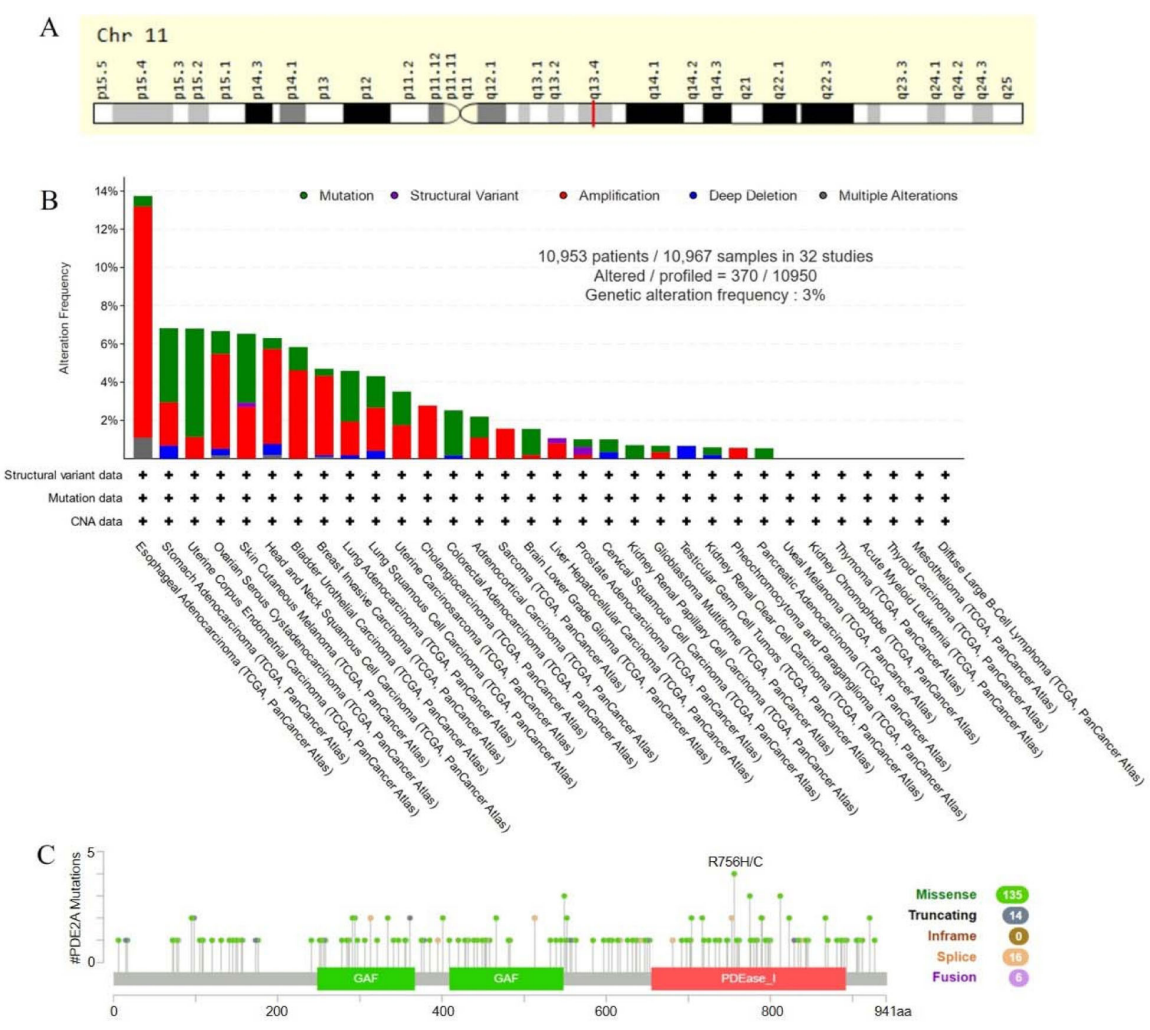
Genomic information and genetic alteration characteristics of PDE2A. **A**, Genomic location of PDE2A gene. **B**, Mutation types and frequencies of PDE2A. **C**, Mutation sites of PDE2A protein domains in pan-cancer.

Numerous studies have discovered that genetic mutations increase the risk of cancer (21). Consequently, we applied GeneCards and cBioportal to get the genomic information and genetic alteration characteristics of PDE2A. PDE2A was a protein-coding gene located at q13.4 in chromosome 11 (Figure 6A). Based on TCGA database, we analyzed mutations in 10,967 samples out of 32 studies. The overall mutation rate of PDE2A genes was approximately 3%, with the major mutation types being Amplification, Mutation, and Deep Deletion. PDE2A mutations were most common in ESAC, STAD, and UCEC (Figure 6B). In addition, among the different types of genetic alteration, the missense mutation was predominant. Moreover, R756H/C was the most frequent mutation site (Figure 6C).

### Immune cell infiltration and TME assessment

**Figure 7:**
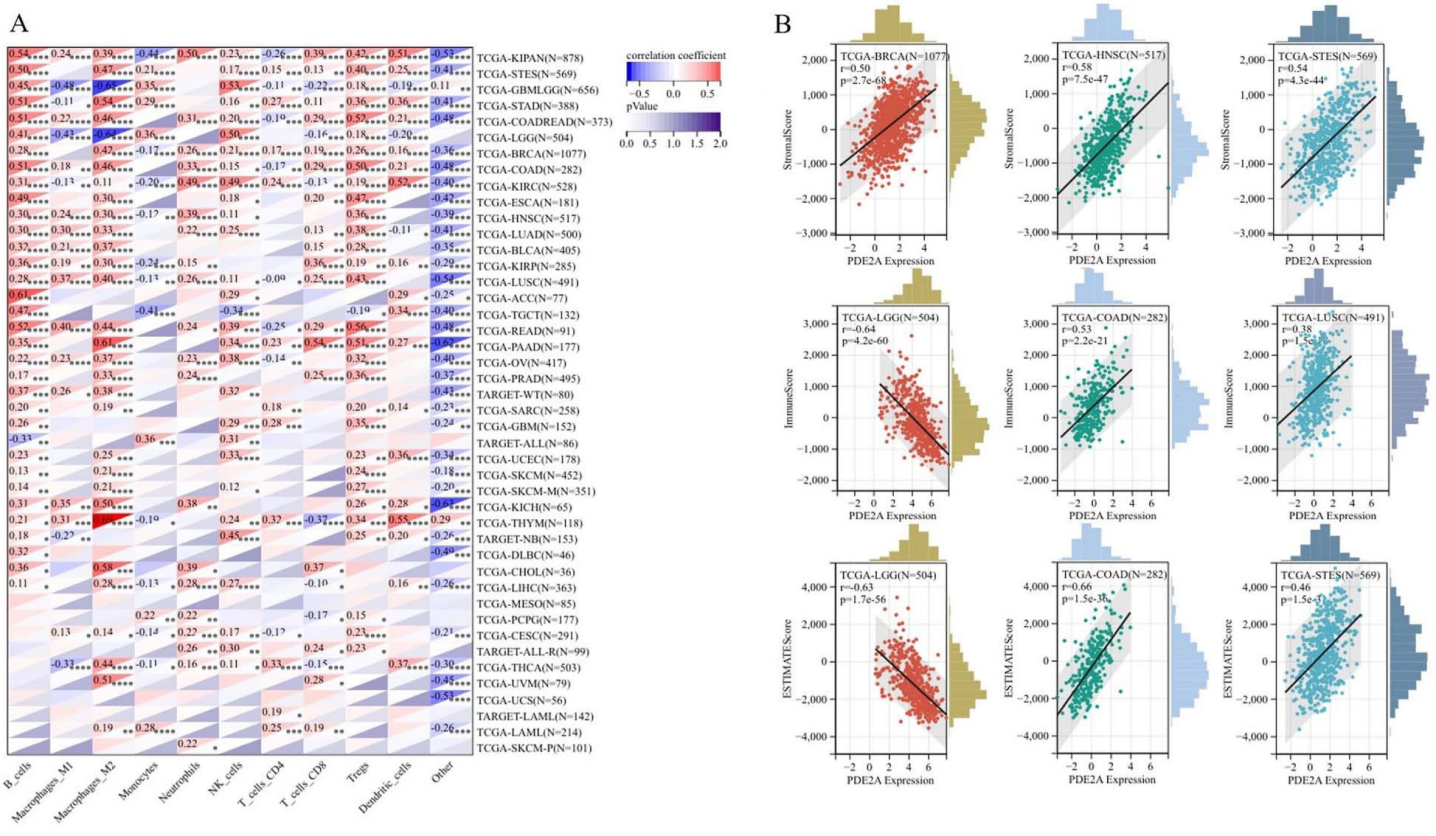
The association between the expression of PDE2A and immune cell infiltration. **A**, Correlation of PDE2A expression with immune infiltration across 44 cancers in TCGA data. *P<0.05, **P<0.01, ***P<0.001, ****P<0.0001 (Spearman's correlation). **B**, The top 3 most significantly correlated cancers with the association between PDE2A expression and Stromal score, Immune score, and ESTIMATE score.

Given that immune cell infiltration is tightly connected to tumor establishment and progression, we explored the association of PDE2A with immune cell infiltration across 44 tumors by quanTIseq algorithm (11). Our results indicated that in various tumors, such as COAD, KIRP, and LUSC, the expression level of PDE2A is positively correlated with the infiltration of B cells, CD8^+^ T cells, M1 macrophages, NK cells, and dendritic cells (DCs). Furthermore, tumor infiltration levels of M2 macrophages and Treg cells exhibit a significant positive correlation with the expression levels of PDE2A in several types of tumors, including STAD, OV, and COAD. Additionally, in LGG, the expression level of PDE2A shows a significant negative correlation with the tumor infiltration level of M2 macrophages (Figure 7A). Therefore, PDE2A may play a pivotal role in tumor-immune interactions.

In TME, stromal cells and immune cells are two major types of non-tumor components, and they have been acknowledged as significant markers for cancer progression and prognostic assessment (22). The ESTIMATE algorithm was used to calculate the stromal, immune, and ESTIMATE scores of tumor tissues. We discovered a highly significant correlation between PDE2A expression and stromal scores specifically in BRCA, HNSC, and STES. In terms of immune scores, the strongest correlations were observed in LGG, COAD, and LUSC. Meanwhile, the top three most significantly correlated cancers for ESTIMATE scores were LGG, COAD, and STES. Interestingly, most correlations were positive except for LGG (Figure 7B). Overall, these results indicated that PDE2A might be a novel immune-associated target in carcinogenesis by affecting stromal cells and immune cells.

### Analysis of the relationship between PDE2A expression and ICP genes and TMB or MSI in pan-cancer samples

**Figure 8:**
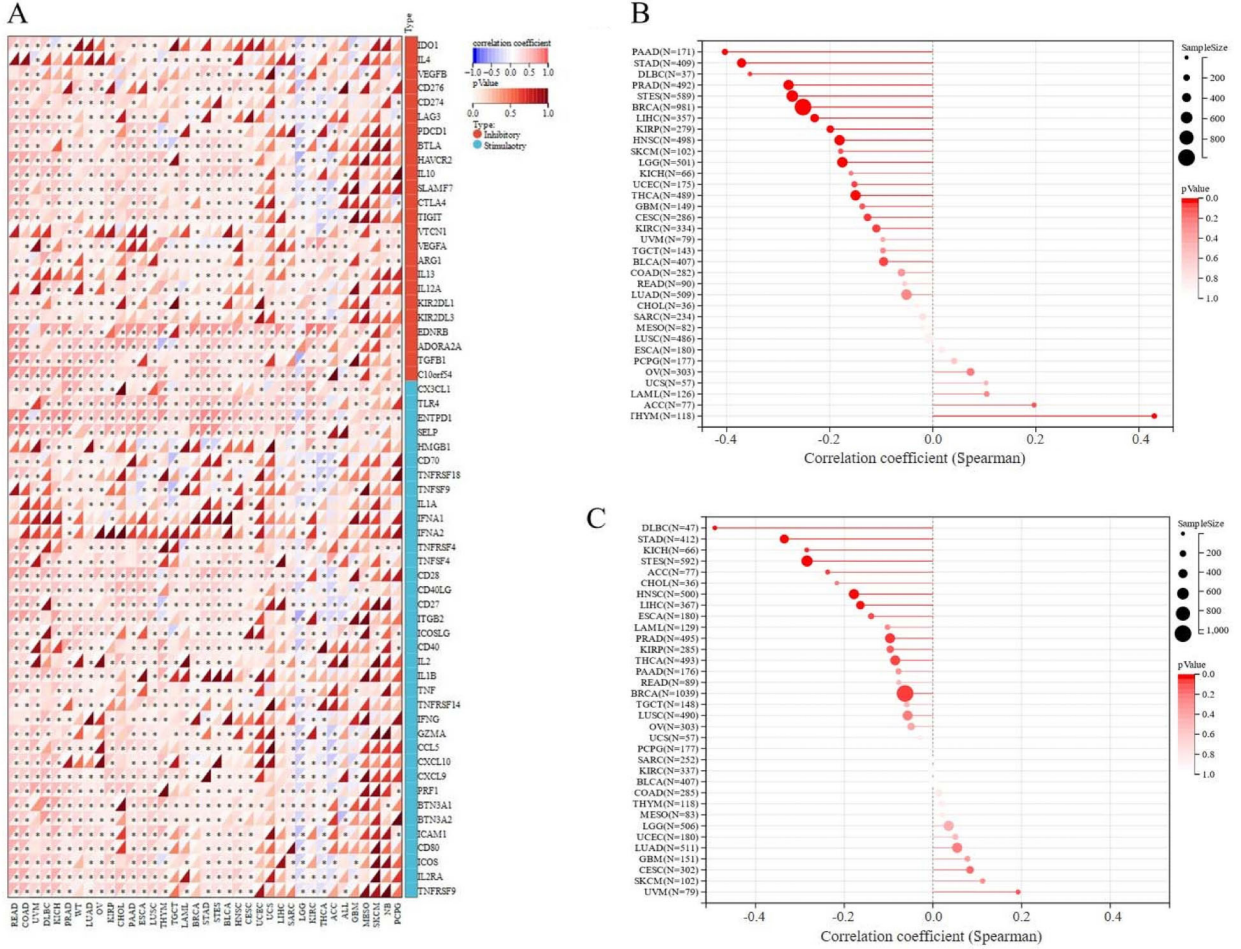
PDE2A and immune checkpoint inhibitors (ICIs) immunotherapy.**A**, Correlation between PDE2A and various immune checkpoint (ICP) genes. **B**, Correlation between PDE2A expression and tumor mutation burden (TMB). **C**, Correlation between PDE2A expression and microsatellite instability (MSI). The Spearman's correlation test was used for statistical analyses.

Immune checkpoint inhibitors (ICIs) therapy is a novel type of immunotherapy, which has been a breakthrough for patients with advanced tumors (23). We investigated the correlation between PDE2A expression and sixty ICP genes to explore the potential of PDE2A in ICIs immunotherapy. As depicted in Figure 8A, PDE2A was significantly positively correlated to the majority of ICP genes in most of human tumors, such as READ, COAD, PRAD, LUAD, LUSC, and STES. In contrast, in LGG, PDE2A was significantly negatively correlated with most of ICP genes. Furthermore, PDE2A expression in most cancers was positively associated with PDCD1, EDNRB, ADORA2A, TGFB1, and especially C10orf54. These results suggested a potential role for PDE2A in modulating the immune response in cancers by targeting these ICP genes.

A higher level of TMB or MSI is associated with better ICI outcomes (24-26). Therefore, we further analyzed the correlations between PDE2A expression and TMB/MSI. As shown in Figure 8B, PDE2A expression had significant negative correlations with TMB in PAAD, STAD, PRAD, STES, BRCA, LIHC, KIRP, HNSC, LGG, THCA (P<0.001), lymphoid neoplasm diffuse large B-cell lymphoma (DLBC), and CESC (P<0.05), while positively correlated in THYM (P<0.001). In addition, the results of correlation analysis showed significant negative associations with MSI in DLBC, STAD, STES, HNSC, LIHC (P<0.001), KICH, ACC, PRAD, and BRCA (P<0.05) (Figure 8C). Based on the above results, it could be speculated that PDE2A might be a good predictive marker for the therapeutic efficacy of ICIs immunotherapy.

### Expression pattern of PDE2A at the single-cell level

**Figure 9:**
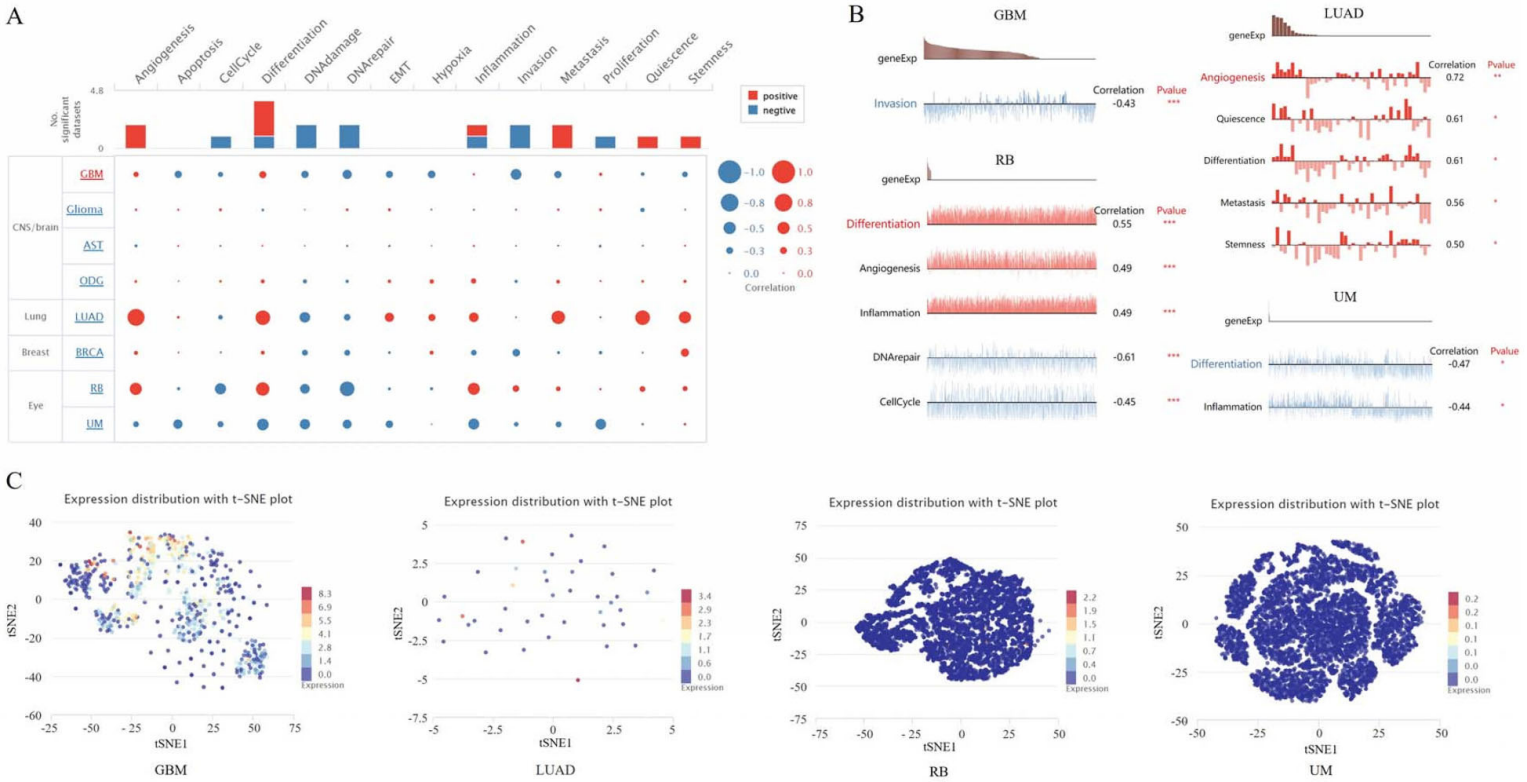
Expression pattern of PDE2A at single-cell levels. **A**, Average correlations between PDE2A expression and functional states in different cancers. **B**, Functional relevance of PDE2A in GBM, LUAD, RB, and UM. *P<0.05, **P<0.01, ***P<0.001 (Student's*t*-test). **C**, T-distributed stochastic neighbor embedding (T-SNE) plots of PDE2A expression at single cells from GBM, LUAD, RB, and UM.

To unravel the biological functional state of PDE2A at the single-cell level in different cancers, we utilized CancerSEA tool. This enabled us to analyze the correlation between PDE2A and functional status of various cancers. As shown in Figure 9A, PDE2A displayed a predominantly positive association with angiogenesis, differentiation, metastasis, quiescence, and stemness. Conversely, the associations between PDE2A and DNA damage, DNA repair, invasion, proliferation, and cell cycle were predominantly negative. In addition, we examined the relationship between PDE2A and functional states in specific cancers. It exhibited a negative correlation with invasion in GBM. In retinoblastoma (RB), PDE2A showed positive correlations with differentiation, angiogenesis, and inflammation. Conversely, PDE2A was negatively correlated with DNA repair and cell cycle. In LUAD, PDE2A was positively correlated with angiogenesis, quiescence, differentiation, metastasis, and stemness. In uveal melanoma (UM), PDE2A was negatively correlated with differentiation and inflammation (Figure 9B). Furthermore, PDE2A expression distribution in GBM, LUAD, RB, and UM were shown at single cell levels by T-SNE diagram (Figure 9C). These results highlighted that PDE2A could be potentially crucial in the development of cancer progression.

### Gene enrichment analysis

**Figure 10:**
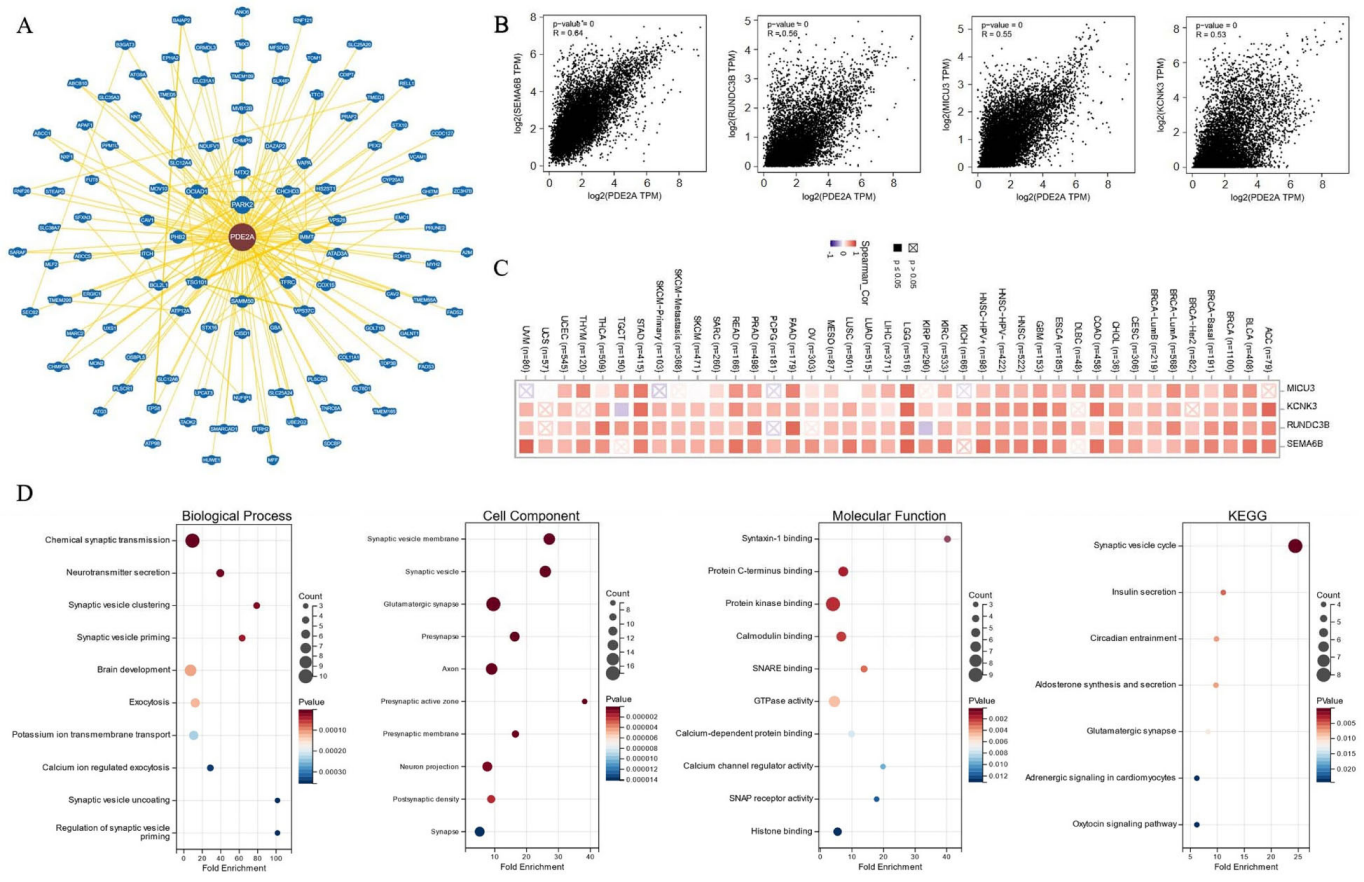
Enrichment analysis of PDE2A-related genes. **A**, PDE2A-related proteins from BioGRID database. **B**, Expression correlation between PDE2A and *SEMA6B*, *RUNDC3B*, *MICU3*, and *KCNK3* genes. P<0.001. **C**, Heatmap of correlation between PDE2A expression and *SEMA6B*, *RUNDC3B*, *MICU3*, and *KCNK3* genes. **D**, The GO/KEGG analysis is based on PDE2A-related genes (Student's *t*-test).

To delve deeper into the molecular mechanisms of PDE2A in oncogenesis and development, we applied PDE2A-correlated genes for functional enrichment analysis. BioGRID was used to obtain the PDE2A-interacted molecules (Figure 10A). Moreover, among the top 100 PDE2A-associated genes downloaded from GEPIA2.0, the top four genes were Semaphorin-6B (*SEMA6B*) (R=0.64), RUN domain containing 3B (*RUNDC3B*) (R=0.56), calcium uptake protein 3 (*MICU3*) (R=0.55), and potassium channel subfamily K member 3 (*KCNK3*) (R=0.53) (all P-values <0.001), which exhibited high correlations with PDE2A across most cancers (Figure 10B). The correlation heatmap showed that PDE2A was positively associated with *SEMA6B*, *RUNDC3B*, *MICU3*, and *KCNK3* genes in the majority of cancers (Figure 10C). Furthermore, based on the GO enrichment analysis, it suggested that PDE2A-related genes were mainly involved in synaptic transmission, such as chemical synaptic transmission, neurotransmitter secretion, and synaptic vesicle clustering. The results of KEGG pathway analysis revealed that synaptic vesicle cycle was predominately associated with the effects of PDE2A on tumor development (Figure 10D).

### Drug sensitivity of PDE2A

We examined the correlation between PDE2A mRNA expression and drug sensitivity using RNAactDrug database. The results showed that increased PDE2A expression was positively related to drug sensitivity of Panobinostat, Sorafenib, Lapatinib, Irinotecan, Topotecan, Nilotinib, PD-0332991, Docetaxel, and Temsirolimus. Conversely, high PDE2A expression was negatively linked to the drug sensitivity of Ruxolitinib, 5-Fluorouracil, and Alectinib (Table 4). These findings suggested that PDE2A expression level may be related to drug resistance of certain chemotherapeutic agents.

**Table 4:** Drug sensitivity of PDE2A.

Compound	RNA type	Source	Spearman.Stat	Spearman.Fdr	P Value	FDA status
Panobinostat	mRNA	CCLE	0.222	5.61×10^-4^	1.48×10^-4^	FDA approved
Sorafenib	mRNA	CCLE	0.216	2.46×10^-3^	2.37×10^-4^	FDA approved
Lapatinib	mRNA	CCLE	0.187	2.31×10^-2^	1.52×10^-3^	FDA approved
Irinotecan	mRNA	CCLE	0.177	9.31×10^-3^	2.66×10^-3^	FDA approved
Topotecan	mRNA	CCLE	0.174	1.05×10^-2^	3.24×10^-3^	FDA approved
Nilotinib	mRNA	CCLE	0.167	4.40×10^-2^	4.67×10^-3^	FDA approved
PD-0332991	mRNA	CCLE	0.164	1.62×10^-2^	5.35×10^-3^	FDA approved
Docetaxel	mRNA	GDSC	0.126	3.42×10^-4^	9.36×10^-5^	FDA approved
Temsirolimus	mRNA	GDSC	0.126	1.10×10^-3^	9.44×10^-5^	FDA approved
Ruxolitinib	mRNA	GDSC	-7.19×10^-2^	4.19×10^-2^	2.68×10^-2^	FDA approved
5-Fluorouracil	mRNA	GDSC	-7.63×10^-2^	3.09×10^-2^	1.87×10^-2^	FDA approved
Alectinib	mRNA	GDSC	-8.39×10^-2^	1.79×10^-2^	9.67×10^-3^	FDA approved

FDA: Food and Drug administration.

## Discussion

In recent years, identifying unique gene expression patterns among different cancer types has provided a promising path for exploring innovative therapeutic targets (27). Through high-throughput sequencing and bioinformatics methods, researchers have conducted in-depth studies on tumor gene expression profiles, which not only help reveal the biological characteristics of tumorigenesis, development, metastasis, and therapeutic resistance but also provide new ideas for molecular typing and individualized treatment of tumors (28). In this comprehensive pan-cancer analysis, we have systematically explored the expression patterns, clinicopathological correlations, prognostic value, genetic and epigenetic alterations, immune cell infiltration, and drug sensitivity associated with the PDE2A gene across multiple human cancers. The results of PDE2A expression in liver cancer (7), osteosarcoma (8), glioblastoma (9), and colorectal cancer (20) were consistent with those reported in prior research. Our findings revealed intricate and diverse roles played by PDE2A in tumorigenesis and cancer progression, highlighting its potential as a therapeutic target and prognostic marker in specific cancer types.

We observed a significant downregulation of PDE2A expression in most types of cancers and across various pathological stages of tumorigenesis, suggesting a potential tumor suppressor role for PDE2A (Figures 2 and 3). However, an intriguing phenomenon was revealed in the survival curve: some tumors exhibited better prognosis when PDE2A expression was high (such as KIRC, LGG, and LIHC), while others showed poorer prognosis (such as BLCA, OV, and STAD), indicating a complex and context-dependent role of PDE2A in cancer progression (Figure 4). The differential prognostic outcomes based on PDE2A expression levels may be attributed to multiple factors, including specific tumor types, tumor heterogeneity, epigenetic modifications, complex gene-gene interactions, tumor microenvironment, and other factors (29). Currently, it remains unclear whether PDE2A exerts similar pathogenic roles across different tumors. The underexpression of PDE2A is associated with poor prognosis in LIHC. Specifically, PDE2A has the potential to regulate the proliferation of hepatocellular carcinoma cells by modulating mitochondrial morphology and ATP content (7). In contrast, favorable prognostic indications were found in OV and STAD. In the progression of OV, PDE2A may exert a potential influence via the Calmodulin/PDE/cAMP/PKA axis pathway (30). Additionally, PDE2A may also play a critical role in the progression of stomach cancer, as evidenced by a previous study demonstrating that PDE2A/GUCY1A1/GUCY1B1 gene expression profiles could distinguish epithelial-mesenchymal transition (EMT)-type gastric tumors from other gastric tumors with high accuracy (31). Further investigation is needed to uncover the possible mechanisms underlying this phenomenon. Together with the results of the Cox regression analyses (Tables 1-3), these findings indicated the prognostic value of PDE2A in LIHC, OV, and STAD. Further high-quality clinical studies are required to validate our findings.

The exploration of DNA methylation patterns revealed intriguing associations between PDE2A methylation and tumorigenesis (Figure 5). The observed decline in PDE2A methylation levels in most cancers suggested epigenetic deregulation as a potential mechanism underlying its altered expression. Conversely, the increased methylation in specific tumor tissues, such as KIRP and PRAD, hinted at a more complex epigenetic landscape that might contribute to the oncogenic phenotypes. These epigenetic modifications could represent promising therapeutic targets for reversing abnormal PDE2A expression in cancers.

DNA methylation is a significant form of epigenetic modification that affects tumor development and biological traits by influencing gene transcription (32). Our study showed that in KIRP, PCPG, PRAD, TGCT, and THCA, the DNA methylation level of PDE2A was higher compared to normal tissues, which inhibited the transcription of PDE2A and resulted in decreased expression levels, consistent with previous research findings. However, the observed decline in PDE2A methylation levels in most cancers suggested the existence of a more complex mechanism for gene expression regulation (33). Although the methylation level of the PDE2A gene was relatively low in tumor tissues, this did not always imply an increase in gene expression. Gene expression is influenced by a variety of other factors, including transcription factor activity, microRNA regulation, the actions of RNA-binding proteins, changes in chromatin structure, intercellular interactions, and the tumor microenvironment (34,35). Therefore, even with reduced methylation, PDE2A gene expression might remain low. To fully understand this phenomenon, further research was necessary to explore the regulatory mechanisms of PDE2A in different tumor types.

Genetic alteration analysis provided insights into the mutational landscape of PDE2A across different cancers (Figure 6). The predominance of missense mutations and the identification of R756H/C as the most frequent mutation site offered valuable information for understanding the functional consequences of these mutations and their potential impact on PDE2A's role in tumorigenesis.

Immune cell infiltration and TME analysis revealed significant associations between PDE2A expression and various immune cell types and stromal cells (Figure 7). Studies have shown that CD8^+^ T cells, B cells, M1 macrophages, NK cells, and DCs are associated with good prognosis in most cancers. Conversely, Treg cells and macrophages (especially M2 macrophages) may indicate poor prognosis (36,37). In STAD, PDE2A expression exhibited a positive correlation with Treg and M2 macrophages infiltration, and a negative correlation with M1 macrophages infiltration. Low PDE2A expression suggested decreased infiltration of Treg and M2 macrophages, increased presence of M1 macrophages, potentially indicating a favorable prognosis for patients. Similarly, in OV, low expression of PDE2A may be more inclined to inhibit the infiltration of Treg and M2 macrophages, resulting in a favorable tumor prognosis. In LIHC, PDE2A expression positively correlated with B cells, NK cells, and DCs. Low PDE2A expression may decrease these immune cell levels, leading to a poor prognosis. This finding suggested a potential role for PDE2A in fostering an immune-active tumor microenvironment in these cancers, which could have significant implications for immunotherapy strategies.

The correlation analysis between PDE2A expression and ICP genes, TMB, and MSI provided further evidence of PDE2A involvement in immune responses and its potential as a biomarker of immunotherapy response (Figure 8). The positive correlations observed between PDE2A and most ICP genes in several cancers suggest a role in enhancing antitumor immunity, while the negative correlations in LGG indicate a possible immunosuppressive function in this specific tumor type. Notably, PDE2A expression was positively correlated with PDCD1 in most tumor tissues. The PDCD1 gene encodes PD-1, a crucial immune checkpoint protein that serves as a significant immunotherapy target in cancer treatment (38). Our research indicated a potential interaction between PDE2A and PD-1, positioning PDE2A as a pivotal mediator that could impact cancer prognosis and tumor immunity. Moreover, higher TMB correlates with better treatment outcomes (39). A recent real-world study has recommended that clinicians consider administering immunotherapy to patients with high TMB, independent of their histological characteristics (26). In our study, PDE2A expression had significant negative correlations with TMB in multiple cancers, including PAAD, STAD, PRAD, STES, BRCA, LIHC, KIRP, HNSC, LGG, THCA, DLBC, and CESC. These findings suggested that PDE2A expression levels could potentially be used as a predictive marker for the therapeutic efficacy of ICIs immunotherapy, especially in cancers where a high TMB was associated with better treatment outcomes. Besides, MSI is known to be a predictor of response to immunotherapy (25). The negative associations between PDE2A and MSI in several tumor types, including DLBC, STAD, STES, HNSC, LIHC, KICH, ACC, PRAD, and BRCA, further support the potential utility of PDE2A as a predictive marker. The significant associations between PDE2A expression and TMB/MSI highlighted its potential as a biomarker for predicting immunotherapy efficacy and response to immune checkpoint inhibitors.

The single-cell level analysis of PDE2A expression offered insights into its functional state in different cancers (Figure 9). Our findings revealed a predominantly positive association between PDE2A and key cancer functions such as angiogenesis, differentiation, metastasis, quiescence, and stemness. Conversely, PDE2A demonstrated primarily negative associations with DNA damage, DNA repair, invasion, proliferation, and cell cycle. Its associations with various cancer functions, both positive and negative, underscored the complexity of its involvement in tumorigenesis.

Gene enrichment analysis identified key PDE2A-associated genes and pathways involved in tumorigenesis and development (Figure 10). The identification of SEMA6B, RUNDC3B, MICU3, and KCNK3 as top PDE2A-correlated genes provided novel targets for functional validation studies aimed at elucidating the molecular mechanisms underlying PDE2A's role in cancer. The involvement of synaptic transmission and synaptic vesicle cycle pathways suggested a potential role for PDE2A in neuronal signaling processes that might be dysregulated in cancers originating from or metastasizing to the nervous system.

Finally, drug sensitivity analysis revealed interesting associations between PDE2A expression and the efficacy of specific anticancer drugs (Table 4). The positive correlation between PDE2A expression and the drug sensitivity of certain compounds, such as Panobinostat, Sorafenib, and Lapatinib, suggested that PDE2A could serve as a predictive biomarker for response to these treatments. Conversely, the negative correlation with Ruxolitinib, 5-Fluorouracil, and Alectinib, indicated a potential role for PDE2A in mediating resistance to these therapies. These findings have important implications for personalizing cancer treatments based on the levels of PDE2A expression.

Despite the comprehensive analysis presented in this study, there are several limitations to acknowledge. One notable limitation is that the analysis largely relied on publicly available datasets, and utilizing microarrays from a variety of such sources may potentially introduce systematic biases. A relatively small sample size can also contribute to data deviation. Additionally, further fundamental research is essential for elucidating the role of PDE2A at the molecular level. The functional implications of PDE2A for immunotherapy require further validation through extensive preclinical and clinical studies. Although PDE2A shows promise as a tumor marker with potential clinical applications, there is still a considerable distance to traverse before it can be effectively applied in clinical practice.

### Conclusions

Our comprehensive pan-cancer analysis of PDE2A revealed its multifaceted roles in tumorigenesis, progression, and immunological roles across multiple cancer types. The prognostic value, genetic and epigenetic alterations, immune cell infiltration patterns, and drug sensitivity associations identified in this study provided valuable insights for developing novel therapeutic strategies targeting PDE2A. Additionally, these findings have implications for predicting patient responses to specific chemotherapy treatments. Future studies should focus on elucidating the molecular mechanisms underlying PDE2A's functions in cancer, validating these findings in larger cohorts, and exploring its potential as a therapeutic target in preclinical and clinical settings.
